# Diversity and Co-Occurrence Patterns of Soil Bacterial and Fungal Communities of Chinese *Cordyceps* Habitats at Shergyla Mountain, Tibet: Implications for the Occurrence

**DOI:** 10.3390/microorganisms7090284

**Published:** 2019-08-22

**Authors:** Jun-Li Shao, Bei Lai, Wei Jiang, Jia-Ting Wang, Yue-Hui Hong, Fu-Bin Chen, Shao-Qing Tan, Lian-Xian Guo

**Affiliations:** 1 Dongguan Key Laboratory of Environmental Medicine, School of Public Health, Guangdong Medical University, Dongguan 523808, China; 2 Department of Basic Medicine, Guangdong Jiangmen Chinese Medical College, Jiangmen 529000, China

**Keywords:** Chinese *Cordyceps*, *Ophiocordyceps sinensis*, soil physicochemical properties, bacterial community, fungal community, co-occurrence analysis

## Abstract

Chinese *Cordyceps* is a well-known medicinal larva-fungus symbiote distributed in the Qinghai-Tibetan Plateau and adjacent areas. Previous studies have involved its artificial cultivation but commercial cultivation is difficult to perform because the crucial factors triggering the occurrence of Chinese *Cordyceps* are not quite clear. The occurrence of Chinese *Cordyceps* is greatly affected by the soil environment, including the soil’s physicochemical and microecological properties. In this study, the effects of these soil properties on the occurrence of Chinese *Cordyceps* were investigated. The results show that the physicochemical properties, including easily oxidizable organic carbon (EOC), soil organic carbon (SOC), humic acid carbon (HAC), humin carbon (HMC), and pH, might be negatively related to the occurrence of Chinese *Cordyceps*, and soil water content (SWC) might be positively related. Several soil physicochemical parameters (pH, SOC, HMC, HAC, available potassium (APO), available phosphorus (APH), microbial biomass carbon (MBC), and the ratio of NH_4_^+^ to NO_3_^−^ (NH_4_^+^/NO_3_^−^)) and microbial properties interact and mix together, which might affect the occurrence of Chinese *Cordyceps*. Soil microbial community structure was also a possible factor, and a low level of bacterial and fungal diversity was suitable for the occurrence of Chinese *Cordyceps*. The intra-kingdom network revealed that a closer correlation of the bacterial community might help the occurrence of Chinese *Cordyceps*, while a closer correlation of the fungal community might suppress it. The inter-kingdom network revealed that the occurrence rate of Chinese *Cordyceps* might be negatively correlated with the stability of the correlation state of the soil habitat. In conclusion, this study shows that soil physicochemical properties and microbial communities could be greatly related with the occurrence of Chinese *Cordyceps*. In addition, soil physicochemical properties, the level of bacterial and fungal diversity, and correlations of bacterial and fungal communities should be controlled to a certain level to increase the production of Chinese *Cordyceps* in artificial cultivation.

## 1. Introduction

*Ophiocordyceps sinensis*, also known as *Cordyceps sinensis*, is a well-known symbiote of fungus (*O. sinensis*) and larva (*Thitarodes*, *Hepialidae*, *Lepidoptera*) [1]. It is particularly distributed in the Qinghai-Tibetan Plateau and adjacent high-altitude areas [2] and is usually called Chinese *Cordyceps* for the fungus-larva symbiote. In this paper, in order to avoid misunderstanding, we use Chinese *Cordyceps* to refer to the fungus-larva symbiote and *O. sinensis* to refer to the fungus. Chinese *Cordyceps* is an important traditional Chinese medicine that can efficiently treat cancer, hyperglycemia, hypertension, and other diseases [3]. These medicinal functions create a large demand for wild Chinese *Cordyceps* [4], but the yield is extremely limited due to its complicated life cycle [5], obligate parasitism, and ecogeographical preference [6]. Worse still, the yield of wild Chinese *Cordyceps* sharply decreased in recent years due to excessive excavation, habitat destruction, and global warming [7]. The serious imbalance between demand and supply leads to soaring retail prices [8]. For decades, increasing studies have focused on artificial cultivation on a large scale, but it has not been widely implemented due to many unanswered questions regarding the mechanism of occurrence (formation of the fungus-larva symbiote) on the host insects [3,9].

**Figure 1:**
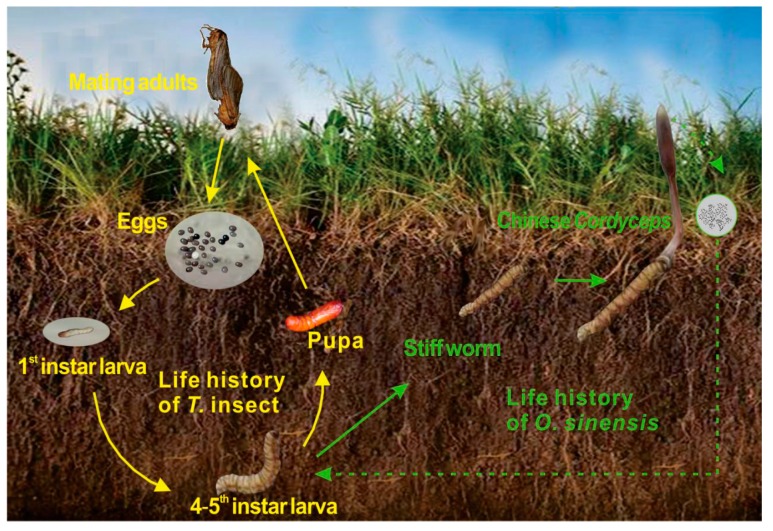
Life history of *Ophiocordyceps sinensis* and *Thitarodes* host [5].

The infection of host *Thitarodes* larvae mainly occurs in soils (Figure 1). *Thitarodes* lives in soil during its larval stage (about 3 years) and pupal stage (40 days) [3]. The fungal spores of *O. sinensis* that erupt from the mature stroma of Chinese *Cordyceps* randomly scatter in topsoils, gradually infiltrate into deeper soils by the delivery of rainfall, develop into infective conidia, and enter into the *Thitarodes* larvae [9]. However, increasing research has proved that the presence of *O. sinensis* might not be the crucial factor in the occurrence of Chinese *Cordyceps*. For instance, the inoculation of *O. sinensis* through spraying, feeding, and injection could not increase the occurrence of Chinese *Cordyceps* very much [10]. Previous reports have also shown that *O. sinensis* existed not only in healthy *Thitarodes* larvae, but also in the egg, pupa, and adult. In addition, several studies have reported that the synergistic effect of different fungi might help to produce Chinese *Cordyceps* [9,11], indicating that the other microbial factors might also be related to its occurrence. Field investigations revealed that although the presence of Chinese *Cordyceps* and the host insect are characterized by a very clear zonal and vertical distribution pattern [12], the occurrence rates (ratio of the number of Chinese *Cordyceps* to the number of *Thitarodes* larvae) show differences within the same region [13]. These discoveries indicated that the occurrence of Chinese *Cordyceps* might be influenced by specific environmental factors, especially soil factors.

The germination and growth of a fungus can be suppressed by natural soils to a certain extent, and this phenomenon is referred to as soil fungistasis [14]. The intensity of fungistasis is dependent on the soil physical and chemical properties as well as microbial activity [15,16]. Among them, the soil microbial community and activity are influenced by the physicochemical characteristics [17], and in turn, the soil microbiome also plays an important role in biogeochemical processes, such as nitrogen, phosphorus, and other element cycles, and is a key factor for soil health and productivity [18]. Thus, it is speculated that the soil microenvironment is closely related to the survival of *O. sinensis* and the occurrence of Chinese *Cordyceps*.

Furthermore, some soil microbes are also influenced by coexisting soil microbes in complicated interaction systems [19,20]. For example, for arbuscular mycorrhizal fungi, fungal spore germination, mycelial growth, and root colonization can be stimulated by some growth factors produced by the mycorrhiza helper bacteria [21]. Thus, research on the relationships among microbial species could aid in understanding interspecies interactions and promote the understanding of the niche spaces among community members [22,23], and these investigations might help to clarify the mechanism of the occurrence of Chinese *Cordyceps*. Yang et al. [24] first attempted to investigate the bacterial communities in the habitats of Chinese *Cordyceps* on the Tibetan Plateau and identified several unique and shared taxa of different soil samples with or without the presence of Chinese *Cordyceps*. Xia et al. [25] reported fungal diversity of the soil adhering to the surface of the membrane covering Chinese *Cordyceps*. However, the comprehensive effects considering soil physicochemical properties and bacterial and fungal communities, especially the network interactions among these properties on the occurrence of Chinese *Cordyceps*, remain insufficient.

In this study, an analysis of the physicochemical properties and the bacterial and fungal communities based on HiSeq sequencing of 16S rRNA and internal transcribed spacer (ITS) genes of the habitat soil of Chinese *Cordyceps* was performed. The purpose of this study was to investigate and explore the influences of soil physicochemical properties and microbial properties and the correlations among these properties on the occurrence of Chinese *Cordyceps*.

## 2. Materials and Methods

### 2.1. Field Site Description and Sample Collection

Chinese *Cordyceps* generally occur in the Qinghai-Tibetan Plateau with the altitudes of higher than 3,500 m, and in this study, native habitats of Chinese *Cordyceps* at Shergyla Mountain, Tibet, were selected as the study region. From 2006 to 2016, a pre-survey was conducted through field investigation on the density of *Thitarodes* larvae and Chinese *Cordyceps*, and the occurrence rate of Chinese *Cordyceps* of each study area was accordingly assessed. Field investigation revealed that the peaks of activity, feeding, growth, development, and population density of *Thitarodes* larvae usually occurred in June to August every year, especially around mid-July, which is coincidently the time for the eruption of ascospores from mature stroma of Chinese *Cordyceps*. Furthermore, during these months, the *Thitarodes* larvae preferred to be situated at the soil depth of 10–20 cm below the ground.

For this study, three sites (each with the area of about 6.6 hectares) with different occurrence rates of Chinese *Cordyceps* were ultimately selected: these sites were named A, B, and C, and the detailed information is presented in Table 1. Briefly, site A had a high density of *Thitarodes* larvae and Chinese *Cordyceps* (occurrence rate of Chinese *Cordyceps*: 10.0%), site B had a high density of *Thitarodes* larvae and low density of Chinese *Cordyceps* (occurrence rate of Chinese *Cordyceps*: 1.4%), and site C had a high density of *Thitarodes* larvae and no Chinese *Cordyceps* (occurrence rate of Chinese *Cordyceps*: 0). The yearly variations of the occurrence rates remained relatively stable at each site, with relative standard deviations (RSD) at site A and B of less than 10%, and null Chinese *Cordyceps* was observed at site C for ten years. At each site, five soil samples were sampled by the diagonal line five-point method in mid-July 2016. During the sampling, weeds on the ground were removed first, and then the soil was cut into v-shaped pits with a shovel. The soil slices of 10–20 cm deep and 10 cm wide were specially sampled. The freshly-collected samples were kept at −20 °C in a portable refrigerator and transported to the laboratory. In the laboratory, roots, plant residues, and stones in the soil samples were removed by sieving through a 2 mm mesh. Each soil sample was divided into 2 parts for DNA extraction and analysis of soil physicochemical properties. The soil samples were stored in −80 °C before the analysis.

### 2.2. Analysis of Soil Physicochemical Properties 

The soil physicochemical properties analyzed in this study included soil water content (SWC), pH, NH_4_^+^-N, NO_3_^−^-N, available phosphorus (APH), available potassium (APO), total nitrogen (TN), easily oxidizable organic carbon (EOC), microbial biomass carbon (MBC), soil organic carbon (SOC), dissolved organic carbon (DOC), extractable humus carbon (HEC), fulvic acid carbon (FAC), humic acid carbon (HAC), and humin carbon (HMC). The physicochemical characteristics were analyzed based on the book *Soil Analysis in Agricultural Chemistry* (in Chinese) [26]. Briefly, SWC was determined by drying at 105 °C for 24 h, soil pH was determined in a soil-water suspension (1:2.5 w/v) by a Corning 610A pH meter (Corning Inc., Corning, NY, USA), NH_4_^+^-N content, NO_3_^−^ content, TN, APH, and APO were determined by a continuous flow analyzer (SAN++, Skalar, Breda, Holland) by testing the soil filtrates after the soil was extracted with 2 M KCl, 0.5 M NH_4_^+^OAc (ammonium acetate, pH = 7), and 1 M NaHCO_3_ (pH = 8.5), EOC was determined by the KMnO_4_ method, SOC was measured by the dichromate method, DOC was determined by analyzing the filtrates of the soil extraction by water, and MBC was determined by the chloroform fumigation-incubation method. The soil humic substance composition, including HEC, FAC, HAC, and HMC, was analyzed according to Xu et al. [27]. The content of HEC, FAC, and HAC was determined by a C/N analyzer (Vario-Max CN analyzer, Elementar Analysensysteme, Hanau, Germany), and the FAC was calculated by subtracting HAC from HEC.

### 2.3. DNA Extraction, PCR, MiSeq Sequencing, and Sequence Data Analysis

Total DNA was extracted from 0.5 g of soil using a MO BIO PowerSoil^®^ DNA Isolation Kit (MO BIO Laboratories Inc., Carlsbad, CA, USA) according to the manufacturer’s instructions. Pipetting and DNA purification were performed on the Microlab^®^ STAR line workstation (Hamilton, Bonaduz, Switzerland) and KingFisher Flex purification system (Thermo Fisher Scientific, Vantaa, Finland). The purified DNA was diluted with 100 μL of RNA-free water (TaKaRa, Dalian, China) and stored at −20 °C for further analysis. The DNA concentrations were measured by a NanoDrop ND−3300 spectrophotometer (NanoDrop Technologies, Thermo Scientific, Wilmington, DE, USA).

The V4 region of 16S rDNA gene and the ITS2 region of the fungal ITS gene were used as the bacterial-specific fragment and fungal-specific fragment respectively, and the primer pairs 515F/806R and ITS3/ITS4 were used to amplify bacterial-specific and fungal-specific fragments, respectively. These primer pairs were modified by a 12-bp barcode sequence at the 5′-end to identify each sample. All amplifications were performed in 50 μL reactions containing 0.5 units of Ex Taq DNA polymerase (TaKaRa, Dalian, China), 10 μL 1× Ex Taq loading buffer (TaKaRa, Dalian, China), 8 μL dNTP mix (TaKaRa, Dalian, China), 2 μL of each primer (10 mM), and 10–100 ng template DNA by an ABI GeneAmp^®^ 9700 PCR System (Applied Biosystems, Waltham, MA, USA). The PCR conditions for bacterial-specific fragments were as follows: 3 min of initial denaturation step at 95 °C, followed by 35 cycles at 94 °C for 30 s, 55 °C for 1 min, and 72 °C for 1 min, and the extension step at 72 °C for 10 min. The PCR conditions for fungal-specific fragments were as follows: 5 min of initial denaturation at 95 °C, followed by 30 cycles at 95 °C for 30 s, 52 °C for 30 s, and 72 °C for 45 s, and the extension step at 72 °C for 10 min. Each sample was amplified 3 times and the products were mixed. After evaluated by 2% agarose gel, the mixed products were purified by an E.Z.N.A.^®^ Gel Extraction Kit (Omega Bio-tek, Norcross, GA, USA) and quantified with a QuantiFluor TM-ST fluorometer (Promega, Madison, WI, USA). In the end, the PCR products were pooled at equimolar concentrations and sequenced on an Illumina MiSeq PE300 platform at Ozimeks Biotech Co., Ltd. (Shenzhen, Guangdong, China). The obtained sequence data were uploaded to the National Center for Biotechnology Information Sequence Read Archive (NCBI SRA) (PRJNA533894). 

The sequence data were further analyzed by QIIME (v. 1.9.0; http://qiime.org/), and the UPARSE pipeline [28] was used for taxonomic assignment with similarities higher than 97%. The taxonomic classification was carried out by the SILVA (v. 119; http://www.arb-silva.de) and UNITE (v. 7.0; http://unite.ut.ee/index.php) databases for bacteria and fungi, respectively. The operational taxonomic unit (OTU) and its tags, which were annotated as chloroplasts or mitochondria (16S amplicons) and cannot be annotated to the kingdom level, were removed, then the OTU taxonomy synthesis information table for the final analysis was generated (Supplementary Table S1). All samples were subsequently subsampled based on the minimum soil microbial sequencing depth in the current study to preclude bias from several sequencing depths.

### 2.4. Statistical Analysis

The statistical analysis of soil physicochemical properties was performed with SPSS (v. 21.0; https://www.ibm.com/analytics/spss-statistics-software). QIIME [29] was used to analyze the Shannon, Simpson, and Chao1 bacterial and fungal diversity indices. Heatmaps were analyzed to compare the top 40 classified genera of bacteria and fungi by the gplot package in R (v. 3.5.3; https://www.r-project.org/). Bray–Curtis, weighted and unweighted unifrac beta diversity indexes were calculated by QIIME software, and accordingly, nonmetric multidimensional scaling (NDMS) analysis was performed and visualized by the vegan package of R software to further illustrate the beta-diversity of soil microbial structure. R was also used to calculate the analysis of similarities (ANOSIM), nonparametric multivariate analysis of variance (MANOVA, or Adonis), and multi-response permutation procedure (MRPP) to compare bacterial and fungal community differences among all soil samples with Bray–Curtis distance and 999 permutations in the R vegan package. The linear discriminant analysis (LDA) effect size (LEfSe) algorithm (http://huttenhower.sph.harvard.edu/galaxy/; last access 23 May 2019) [30] was used to identify the taxa that were present in different abundances between the Chinese *Cordyceps* group (sites A and B) and null Chinese *Cordyceps* group (site C). LEfSe emphasizes statistical significance and biological relevance. The effect size threshold of LDA score was set to 3.5 for analysis in this study. To examine the effects of soil physicochemical properties on structuring microbial communities, Mantel tests and redundancy analyses (RDA) were performed, and the results were visualized by the vegan package of R software. To demonstrate the relationships among different microbial species at each site, intra-kingdom network analysis was conducted using the 40 most abundant bacterial and fungal genera, and inter-kingdom network analysis was conducted using different microbial families (including bacterial and fungal families) at each site. Highly significant positive (*R* > 0.80, false discovery rate (FDR) < 0.05) and negative (*R* < −0.80, FDR < 0.05) Pearson correlations were screened out and co-occurrence patterns were visualized as networks using Cytoscape version 3.6.0 (https://cytoscape.org/) [31]. The size and color of each node represented the number of connections and taxonomy, respectively. 

## 3. Results

### 3.1. Soil Physicochemical Properties

The soil physicochemical properties for each site are shown in Table 2. Compared to site A, site B had significantly higher NH_4_^+^-N, NO_3_^−^-N, APH, APO, EOC, MBC, SOC, DOC, HEC, FAC, HAC, and HMC (*p* < 0.05), and had significantly lower NH_4_^+^-N/NO_3_^−^-N. Compared to site A, site C had significantly higher pH, APH, APO, EOC, MBC, SOC, DOC, HEC, HAC, and HMC (*p* < 0.05), and had significantly lower SWC, NH_4_^+^-N and NO_3_^−^-N. Compared to site B, site C had higher pH (*p* < 0.05) and NH_4_^+^-N/NO_3_^−^-N, and lower SWC, NH_4_^+^-N, NO_3_^−^-N, APH, APO, MBC, DOC, HEC, and FAC (*p* < 0.05). 

### 3.2. Soil Microbial Diversity 

Through the raw sequencing reads, there was a total of 602,677 high-quality 16S rDNA sequences and 256,324 high-quality ITS2 sequences. In addition, 10,762 bacterial OTUs and 3361 fungal OTUs were clustered from these high-quality sequences with a 97% identity threshold. Alpha diversity was applied in analyzing complexity of species diversity for a sample through five indices, including two indices to identify community richness: observed species and Chao1, and three indices to identify community diversity: Shannon, Simpson, and Dominance [32]. The alpha diversity indices of bacterial and fungal communities are shown in Table 3. For bacteria, the diversity (represented by Shannon and Simpson indices) of site C was significantly higher than that of site B (*p* < 0.05), and the bacterial richness (represented by Chao 1) of site C was significantly higher than that of sites A and B. For fungi, the diversity (represented by Shannon and Simpson indices) of sites B and C was significantly higher than that of site A (*p* < 0.05).

**Figure 2:**
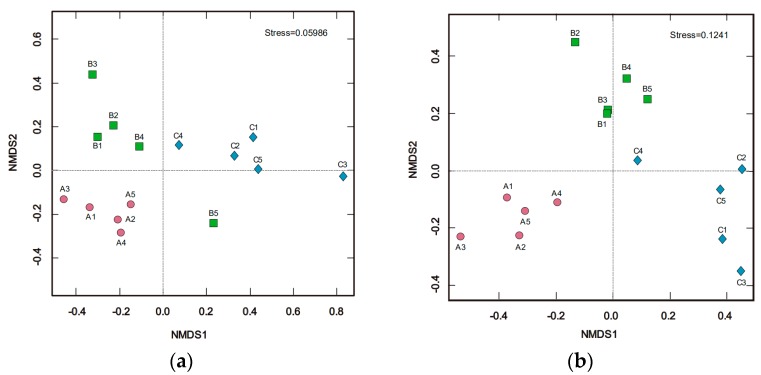
Nonmetric multidimensional scaling (NMDS) based on Euclidean distance plot of all soil (**a**) bacterial and (**b**) fungal communities. Red circles, green squares, and blue diamonds represent samples from sites A, B, and C, respectively.

Beta diversity analysis was used to evaluate differences of samples in species complexity. In this study, beta-diversity at the OTU level by NMDS [33] analysis is shown in Figure 2. According to Euclidean distance dissimilarity, the bacterial (Figure 2a) and fungal (Figure 2b) beta-diversities were different for each site and the samples within each site were clearly grouped together. In addition, significant differences (*p* < 0.05) were found among all soil samples in aspects of bacterial and fungal communities, as shown in Table 4, corresponding to ANOSIM, Adonis, and MRPP analysis.

**Figure 3:**
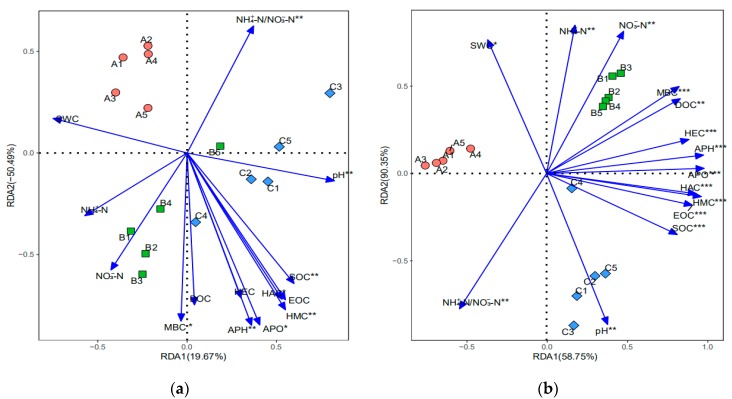
Redundancy analysis (RDA) demonstrating the significant relationships between soil physicochemical factors and (**a**) bacterial and (**b**) fungal communities. Red circles, green squares, and blue diamonds represent samples from sites A, B, and C, respectively. Soil physicochemical factors are represented as sold lines with filled arrows. * *p* < 0.05, ** *p* < 0.01, *** *p* < 0.001.

The influence from soil physicochemical factors on the soil microbial community was examined by Mantel tests (Table S2) and redundancy analysis (RDA), and the significant relationships (*p* < 0.05) between soil physicochemical factors and microbial communities are shown in Figure 3. In Figure 3, soil physicochemical factors are represented by arrows. The length of the arrow line represents the degree of correlation between the physicochemical factor and microbial communities, and the projection distance of each soil sample on the arrow line represents the degree of correlation between the physicochemical factor and the sample [34]. Thus, from Figure 3a, the soil bacterial communities are significantly correlated with NH_4_^+^-N/NO_3_^−^-N, pH, SOC, HAC, HMC, APO, APH, and MBC (*p* < 0.05), among them, NH_4_^+^-N/NO_3_^−^-N, MBC and pH, present the highest correlation with samples from site A, B, and C, respectively. From Figure 3b, the soil fungal communities’ structure are significantly correlated with SWC, NH_4_^+^-N, NO_3_^−^-N, NH_4_^+^-N/NO_3_^−^-N, MBC, DOC, HEC, APH, APO, HAC, HMC, EOC, SOC, and pH (*p* < 0.05), among them, SWC presents the highest correlation with samples from site A, and NH_4_^+^-N, NO_3_^−^-N, MBC, DOC, HEC, APH, and APO present the highest from site B, and pH, SOC, EOC, HAC, HMC, APH present the highest from site C. Overall, NH_4_^+^-N/NO_3_^−^-N, pH, SOC, HAC, HMC, APO, APH, and MBC could significantly affect both the bacterial and fungal community structure. 

### 3.3. Soil Bacterial and Fungal Structure

**Figure 4:**
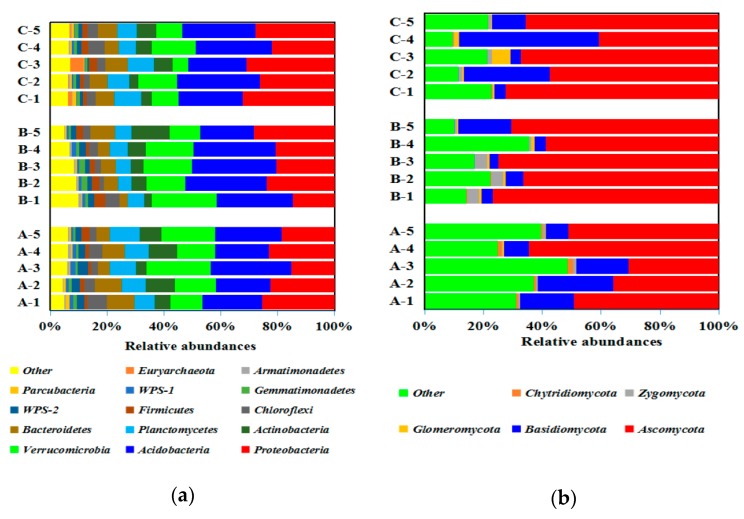
Relative abundance of (**a**) bacterial and (**b**) fungal phyla in different sites. “Other” includes phyla with less than 0.1% of relative abundance and unclassified phyla.

The relative compositions of soil bacterial and fungal communities at the phylum, class, order, family, and genus level are presented in Supplementary Tables S3 and S4, respectively. The predominant phyla (with relative abundance higher than 0.01%) of bacterial communities were *Proteobacteria*, *Acidobacteria*, *Verrucomicrobia*, *Actinobacteria*, *Planctomycetes*, *Bacteroidetes*, *Chloroflexi*, *Firmicutes*, *WPS−2*, *Gemmatimonadetes*, *WPS−1*, *Parcubacteria*, *Armatimonadetes*, and *Euryarchaeota*, occupying more than 90% of the total sequences (Figure 4a). Five predominant phyla (with relative abundance higher than 0.01%) of fungal communities were identified: *Ascomycota*, *Basidiomycota*, *Glomeromycota*, *Zygomycota*, and *Chytridiomycota*, occupying more than 50% of the total sequences (Figure 4b). Besides these five fungal phyla, the proportion of other fungal phyla was especially enriched in site A.

**Figure 5:**
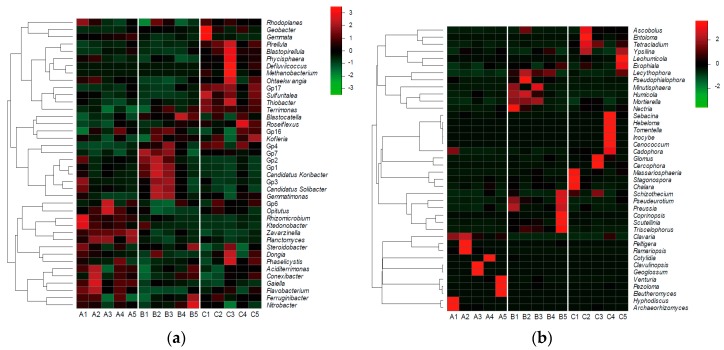
Heatmaps of top 40 genera of soil (**a**) bacterial and (**b**) fungal communities at each site. Relative abundance of soil microbial community from high to low is represented by red, black, and green.

The 40 most abundant genera of soil bacterial and fungal communities can be seen in Figure 5a,b respectively, and the relative abundance of soil microbial community from high to low is represented by red, black, and green. The top genera varied among the sample sites: for bacterial communities, site A had high relative abundance of *Gp6*, *Opitutus*, *Rhizomicrobium*, *Ktedonobacter*, *Aciditerrimonas*, *Conexibacter*, *Gaiella*, and *Flavobacterium*, site B had high relative abundance of *Blastocatella*, *Kofleria*, *Gp4*, *Gp7*, *Gp2*, *Gp1*, *Candidatus Koribacter*, *Gp3*, *Candidatus Solibacter*, and *Gemmatimonas*, and site C had high relative abundance of *Geobacter*, *Gemmata*, *Pirelllula*, *Blastopirellula*, *Defluviicoccus*, *Methanobacterium*, *Ohtaekwangia*, and *Gp17*. For fungal communities, site A had high relative abundance of *Archaeorhizomyces*, *Hyphodiscus*, *Beutheromyces*, *Pezoloma*, *Venturia*, *Geogiossum*, *Clavulinopsis*, *Cotylidia*, *Rammanopsis*, *Peltigera*, and *Cavana*, site B had high relative abundance of *Triscelophorus*, *Scutellinia*, *Coprinopsis*, *Preussia*, *Pseudeurotium*, *Schizothecium*, *Nectria*, *Mortierella*, *Humicola*, *Minutisphaera*, and *Pseudophialophora*, and site C had high relative abundance of *Chalara*, *Stagnonospora*, *Massariosphaeria*, *Cercophora*, *Glomus*, *Cadophora*, *Cenococcum*, *Inocybe*, *Tomentella*, *Hebeloma*, *Sebacina*, *Sebacina*, *Exophiala*, *Leohumicola*, *Ypsilina*, *Tetracladium*, *Entoloma*, and *Ascobolus*.

### 3.4. Differential Operational Taxonomic Units (OTUs) Related to the Occurrence of Chinese Cordyceps

**Figure 6:**
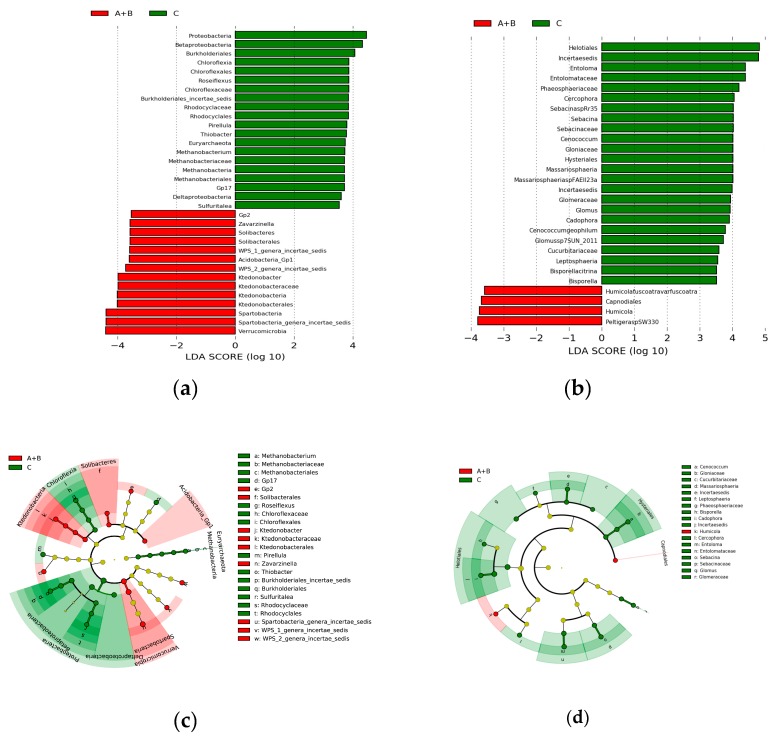
Differentially abundant microbial taxa in Chinese *Cordyceps* group (sites A and B) and null Chinese *Cordyceps* group (site C) illustrated using linear discriminant analysis (LDA) effect size analysis and cladograms for (**a**,**c**) bacteria and (**b**,**d**) fungi. Red represents taxa-enriched in sites A and B, green represents taxa-enriched in site C. Microbial taxa were determined by LDA with a significant threshold over 3.5.

In order to discuss the detailed OTUs that might be related to the occurrence of Chinese *Cordyceps*, the differential OTUs between the Chinese *Cordyceps* group (sites A and B) and the null Chinese *Cordyceps* group (site C) were screened out as biomarkers using linear discriminant analysis (LDA) effect size analysis. The LDA score of these biomarkers was illustrated by the histograms (Figure 6a,b), the length of each bar representing the degree of the differences. The taxonomic information of these biomarkers was illustrated by cladograms (Figure 6c,d), with the circles radiating from the center point representing the taxonomic levels from phylum to species. Fourteen bacterial OTUs (mostly belonging to the classes *Ktedonobacteria*, *Solibacteres*, *Acidobacteria*, and *Verrucomicrobia*) and four fungal OTUs (mostly belonging to *Capnodiales*, *Humicola*, and *PeltigeraspSW330*) presented significantly higher abundance in the soil samples of the Chinese *Cordyceps* group (sites A and B). Twenty bacterial OTUs (mostly belonging to the classes *Methanobacteria*, *Betaproteobacteria*, *Chloroflexia*, *Deltaproteobacteria*, *Planctomycetia*, *Pirellula*, and *Acidobacteria*) and 24 fungal OTUs (mostly belonging to the classes *Leotiomycetes*, *Dothideomycetes*, *Agaricomycetes*, *Sordariomycetes*, *Glomeromycetes*, and *Glomeromycetes*) were significantly enriched in the null Chinese *Cordyceps* group (site C).

**Figure 7:**
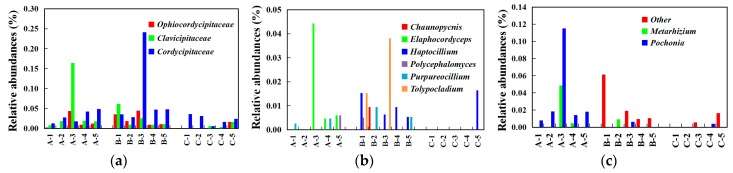
Relative abundance of *Cordyceps*-related fungi in different sampling groups: (**a**) *Cordyceps*-related families, (**b**) *Ophiocordycipitaceae* family, and (**c**) *Clavicipitaceae* family.

In terms of the most concerned *Cordyceps*-related families [35], *Clavicipitaceae*, *Cordycipitaceae*, and *Ophiocordycipitaceae* were identified but in minor abundance in this study: *Cordycipitaceae* appeared in all samples, and *Ophiocordycipitaceae* and *Clavicipitaceae* presented preference in the Chinese *Cordyceps* group (sites A and B) (Figure 7a). Among them, *Metarhizium*, *Pochonia*, *Simplicillium*, *Elaphocordyceps*, *Polycephalomyces*, *Purpureocillium*, and *Tolypocladium* presented preference in the Chinese *Cordyceps* group (Figure 7b,c).

### 3.5. Intra-kingdom Co-Occurrence Analysis

**Figure 8:**
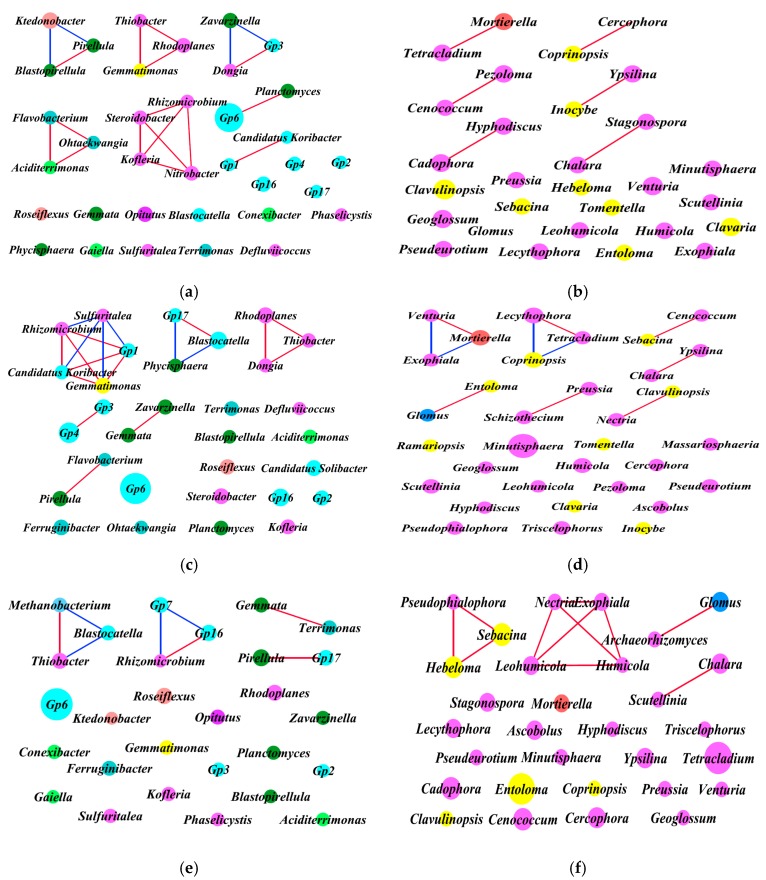
Intra-kingdom network of 40 most abundant co-occurring bacterial and fungal genera based on correlation analysis. Connecting lines represent strong (Spearman’s ρ > 0.6) and significant (*p* < 0.01) correlation. Highly significant positive (*R* > 0.80, false discovery rate (FDR) < 0.05) and negative (*R* < −0.80, FDR < 0.05) Pearson correlations are represented as red and blue lines. The size of each node is proportional to the degree, and nodes are colored by taxonomy. (**a**,**c**,**e**) Bacterial correlation patterns for sites A, B, and C, respectively. ■, ■, ■, ■, ■, ■, ■, and ■ represent *Acidobacteria*, *Actinobacteria*, *Bacteroidetes*, *Chloroflexi*, *Gemmatimonadetes*, *Planctomycetes*, *Proteobacteria*, and *Verrucomicrobia*, respectively. (**b**,**d**,**f**) Fungal correlation patterns for sites A, B, and C, respectively. ■, ■, ■, and ■ represent *Ascomycota*, *Basidiomycota*, *Glomeromycota*, and *Zygomycota*, respectively.

**Figure 9:**
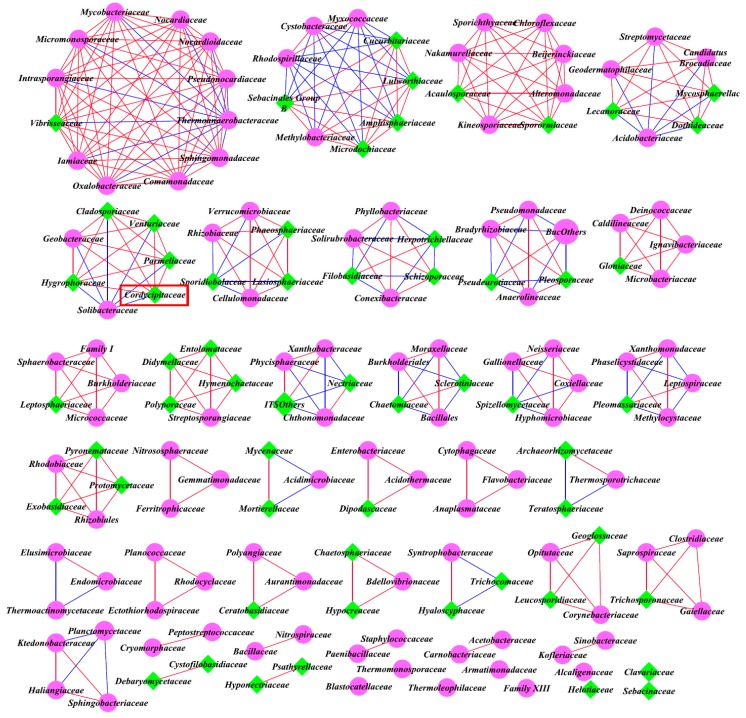
Inter-kingdom network of differentially abundant fungal and bacterial families at site A. Highly significant positive (*R* > 0.80, FDR < 0.05) and negative (*R* < −0.80, FDR < 0.05) Pearson correlations are represented by red and blue lines. The size of each node is proportional to the degree. Fuchsia circles: bacterial kingdoms, green diamonds: fungal kingdoms, red rectangle highlights *Cordyceps*-related family.

**Figure 10:**
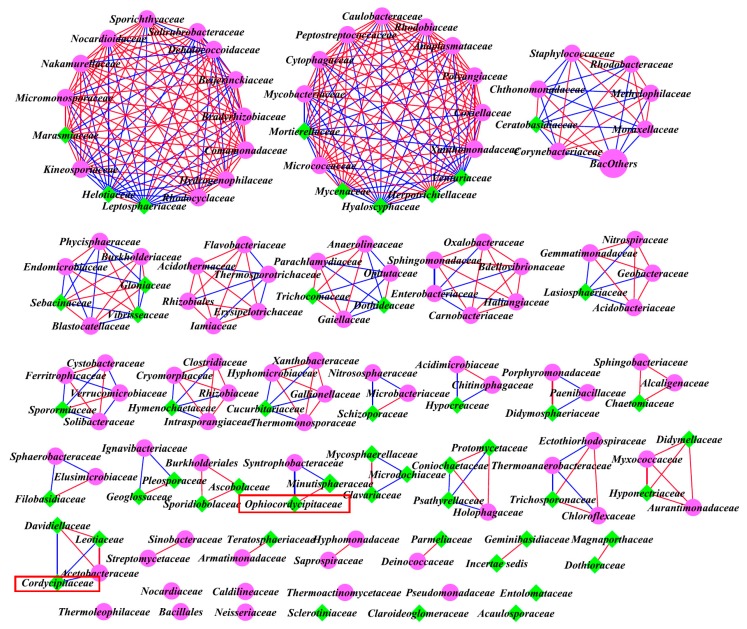
Inter-kingdom network of differentially abundant fungal and bacterial families at site B. Highly significant positive (*R* > 0.80, FDR < 0.05) and negative (*R* < −0.80, FDR < 0.05) Pearson correlations are represented by red and blue lines. The size of each node is proportional to the degree. Fuchsia circles: bacterial kingdoms, green diamonds: fungal kingdoms, red rectangle highlights *Cordyceps*-related family.

**Figure 11:**
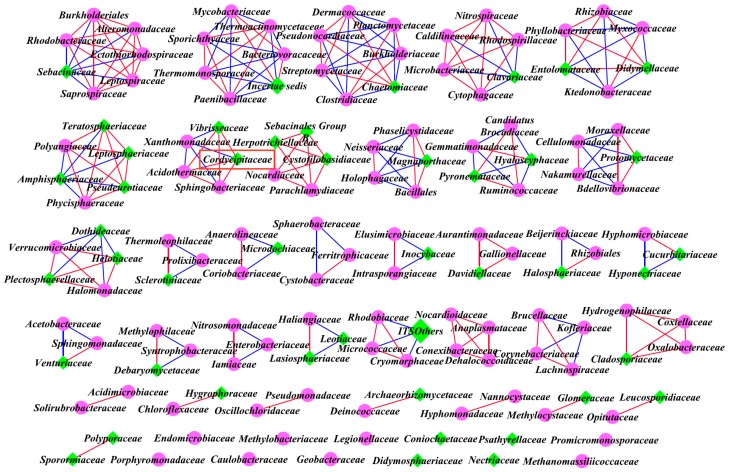
Inter-kingdom network of differentially abundant fungal and bacterial families at site C.Highly significant positive (*R* > 0.80, FDR < 0.05) and negative (*R* < −0.80, FDR < 0.05) Pearson correlations are represented by red and blue lines. The size of each node is proportional to the degree. Fuchsia circles: bacterial kingdoms, green diamonds: fungal kingdoms, red rectangle highlights *Cordyceps*-related family.

Network analysis is widely performed to explore the interactions of microbial taxon in the complex microbial communities. In this study, network analysis was applied to illustrate the differences of soil bacterial and fungal communities among different sampling sites (Figure 8, Figure 9, Figure 10 and Figure 11), and the network properties are summarized in Table 5. In Figure 8, Figure 9, Figure 10 and Figure 11, red and blue lines represented that the abundances between the two connected genera (or families) are positively correlated and negatively correlated, respectively.

Based on the top 40 bacterial and fungal genera, the intra-kingdom network analysis of the correlations is shown in Figure 8 and Table 5. For bacterial analysis (Figure 8a,c,e), the co-occurrence analysis showed 16 positive and 4 negative significant correlations, 13 positive and 6 negative significant correlations, and 4 positive and 4 negative significant correlations at sites A, B, and C, respectively. For fungal analysis (Figure 8b,d,f), the co-occurrence analysis showed 6, 7, and 11 significant positive correlations at sites A, B, and C respectively, and only 4 significant negative correlations at site B, with no significant negative correlations observed at sites A and C. The average degree of bacterial communities was 1.143, 1.256, and 0.571, and fungal communities was 0.414, 0.667, and 0.733 respectively, at sites A, B, and C (Table 5). Hence, there were more positive correlations and closer relationships of bacterial taxa at sites A and B, and they were highest for the fungal taxa at site C.

The “hub” taxa were defined as the most interactive genera (or families) [30,36]. In general, the bacterial hub taxa mostly belonged to the phyla *Acidobacteria*, *Betaproteobacteria*, *Alphaproteobacteria*, and *Planctomycetia*, and the fungal hub taxa mainly belonged to the phyla *Leotiomycetes* and *Sordariomycetes.* Specifically, in the top 40 bacterial communities, the hub taxa were *Nitrobacter*, *Steroidobacter*, *Rhizomicrobium*, and *Kofleria* at site A, *Gemmatimonas*, *Rhizomicrobium*, *Sulfuritalea*, *Gp1*, *Candidatus*, and *Koribacter* at site B, and *Methanobacterium*, *Gp7*, *Blastocatella*, *Thiobacter*, *Gp16*, and *Rhizomicrobium* at site C. For the top 40 fungal communities, the hub taxa were *Tetracladium*, *Coprinopsis*, *Cenococcum*, *Inocybe*, *Cadophora*, *Cercophora*, *Chalara*, *Stagonospora*, *Pezoloma*, *Mortierella*, *Hyphodiscus*, and *Ypsilina* at site A, *Tetracladium*, *Coprinopsis*, *Venturia*, *Lecythophora*, *Mortierella*, and *Exophiala* at site B, and *Exophiala*, *Nectria*, *Humicola*, and *Leohumicola* at site C. 

### 3.6. Inter-kingdom Co-Occurrence Analysis and Determination of Hub Taxa

Among all of the bacterial and fungal families detected in the three sites, highly significant positive (*R >* 0.80, FDR < 0.05) and negative (*R <* −0.80, FDR < 0.05) Pearson correlations were found among all families detected at the three sites. The corresponding network properties are summarized in Table 5 and occurrence patterns are visualized as a network in Figure 9 (site A), Figure 10 (site B), and Figure 11 (site C). Among all families, the co-occurrence analysis showed 257 positive and 95 negative significant correlations, 243 positive and 158 negative significant correlations, and 182 positive and 157 negative significant correlations at sites A, B, and C, respectively. The average degree of sites A, B, and C was 4.241, 5.383, and 3.831 respectively, showing more connections and closer relationships at sites A and B.

The hub taxa were *Iamiaceae*, *Intrasporangiaceae*, *Micromonosporaceae*, *Mycobacteriaceae*, *Nocardiaceae*, *Nocardioidaceae*, *Pseudonocardiaceae*, *Thermoanaerobacteraceae*, *Sphingomonadaceae*, *Comamonadaceae*, *Oxalobacteraceae*, and *Vibrisseaceae* at site A, *Kineosporiaceae*, *Micrococcaceae*, *Micromonosporaceae*, *Mycobacteriaceae*, *Nakamurellaceae*, *Nocardioidaceae*, *Sporichthyaceae*, *Solirubrobacteraceae*, *Cytophagaceae*, *Dehalococcoidaceae*, *Peptostreptococcaceae*, *Caulobacteraceae*, *Beijerinckiaceae*, *Bradyrhizobiaceae*, *Rhodobiaceae*, *Anaplasmataceae*, *Comamonadaceae*, *Hydrogenophilaceae*, *Rhodocyclaceae*, *Polyangiaceae*, *Coxiellaceae*, *Xanthomonadaceae*, *Leptosphaeriaceae*, *Venturiaceae*, *Herpotrichiellaceae*, *Helotiaceae*, *Hyaloscyphaceae*, *Marasmiaceae*, *Mycenaceae*, and *Mortierellaceae* at site B, and *Methanobacteriaceae*, *Blastocatellaceae*, *Chthonomonadaceae*, *Family I*, *Alcaligenaceae*, *Bac Others*, *Phaeosphaeriaceae*, *Strophariaceae*, and *Thelephoraceae* at site C.

In terms of Cordyceps-related fungal families at site A (*Ophiocordycipitaceae*, *Clavicipitaceae*, and *Cordycipitaceae*), *Cordycipitaceae* was positively correlated with *Parmeliaceae*, *Venturiaceae*, *Cladosporiaceae*, *Geobacteraceae*, and *Hygrophoraceae*, and negatively correlated with *Solibacteraceae*, at site B, *Ophiocordycipitaceae* was positively correlated with *Minutisphaeraceae* and negatively correlated with *Syntrophobacteraceae*, and *Cordycipitaceae* was negatively correlated with *Acetobacteraceae*, *Leotiaceae*, and *Davidiellaceae*, at site C, *Cordycipitaceae* was positively correlated with *Vibrisseaceae*, *Acidothermaceae*, and *Sphingobacteriaceae*, and negatively correlated with *Xanthomonadaceae*.

## 4. Discussion

Chinese *Cordyceps* is the outcome of the infection, colonization, and growth of *O. sinensis* on *Thitarodes* larvae. Previous studies revealed that the growth of fungi is dependent on physical, chemical, and microbial properties of soil [15,16]. Thus, the soil microenvironment is closely related to the occurrence rate of Chinese *Cordyceps*. This study revealed that the soil microenvironment is a complex community, and these properties might be correlated or might co-affect the occurrence of Chinese *Cordyceps.*

### 4.1. Soil Physicochemical Properties

Limited studies have reported that soil physicochemical properties, including available K and pH, could affect the occurrence of Chinese *Cordyceps* [12,37]. In this study, it was found that there were significant differences in pH, NH_4_^+^-N, NO_3_^−^-N, APH, APO, MBC, DOC, HEC, DOC, and HEC between site C and site A (or site B) (Table 2). These findings reflect that besides K and pH, various soil physicochemical properties could affect the occurrence of Chinese *Cordyceps*. Nevertheless, the contents of those physicochemical factors did not present progressive increase or decrease of the occurrence rates, i.e., the contents presented A > B > C or A < B < C. For instance: (1) the SWC presented A≈ B > C, (2) the contents of EOC, SOC, HAC and HMC presented A < B ≈ C, (3) pH presented A ≈ B < C, (4) the contents of NH_4_^+^-N and NO_3_^−^-N presented C < A < B while (5) the ratio of NH_4_^+^-N/NO_3_^−^-N presented B < A ≈ C, and (6) APH, APO, MBC, DOC and HEC presented A < C < B. Therefore, the levels of EOC, SOC, HAC, HMC, and pH might significantly inhibit the occurrence of Chinese *Cordyceps*, and the levels of SWC might be positively related with the occurrence of Chinese *Cordyceps*, and the levels of NH_4_^+^-N, NO_3_^−^-N, NH_4_^+^-N/NO_3_^−^-N, APH, APO, MBC, DOC, and HEC might be related to the occurrence of Chinese *Cordyceps*, while a detailed relationship could not be clarified based on the current study. In addition, soil is a rather complicated matrix, and all of the physicochemical factors might co-affect *Thitarodes* larvae, and the occurrence of Chinese *Cordyceps* might be the outcome of the synergy of those factors. These inferences should be further confirmed in future studies.

Physicochemical parameters could affect the bacterial and fungal communities [38,39]. In this study, both Mantel test and RDA demonstrated that the soil physicochemical parameters significantly regulated the soil bacterial communities, including NH_4_^+^-N/NO_3_^−^-N, pH, SOC, HAC, HMC, APO, APH, and MBC (*p* < 0.05), and fungal communities, including SWC, NH_4_^+^-N, NO_3_^+^-N, NH_4_^+^-N/NO_3_^−^-N, MBC, DOC, HEC, APH, APO, HAC, HMC, EOC, SOC, and pH (*p* < 0.05). Thus, these soil physicochemical parameters are important for the structures of the soil microbial community. In addition, the microbial community of the soil samples are scattered in four or three quadrants in Figure 5, and the differences of their correlations with various soil physicochemical factors represent the different degrees of influence from soil physicochemical factors on the microbial community. For the samples collected from site A, the bacterial community presents the highest correlation with NH_4_^+^-N/NO_3_^−^-N, and the fungal community presents higher correlation with SWC than site C. Thus, from the perspective of constructing a soil microbial community conducive to the occurrence of Chinese *Cordyceps*, the ratio of NH_4_^+^-N/NO_3_^−^-N, and the water content of the soil should be in consideration. The current study illustrated that soil physicochemical factors influenced soil bacterial and fungal community structure in different degrees. On the other hand, soil microbial community is also important to the soil ecosystem stability and nutrient transformation, and in turn would affect the soil physicochemical properties [18]. Therefore, soil physicochemical parameters and microbial properties could interact and mix together, which might affect the occurrence of Chinese *Cordyceps*.

### 4.2. Soil Microbial Structure

Besides the interactions between physicochemical and microbial factors, the interactions between different microbial species might also be related to the occurrence. For instance, soil microbial community structure could interact with fungi and affect the soil fungistasis [14]. On the other hand, some fungi-helper microbiota could produce growth factors to stimulate fungal spore germination, mycelial growth, and host colonization, and reduce environmental stress by alleviating the toxification of antagonistic substances and inhibiting competitors and antagonists [21]. Besides *O. sinensis*, some other species were proved to be closely associated with Chinese *Cordyceps* [11] and may be involved in the development of its stromata [40]. Thus, the soil microbial community might be important to the occurrence of Chinese *Cordyceps*. In the current study, it was found that the bacterial diversity of sites A and B were significantly less than that of site C, and the fungal diversity of site A was significantly less than that of sites B and C (Table 3). As the occurrence of Chinese *Cordyceps* was highest in site A, these findings indicate that the decreased microbial diversity was an advantage for the occurrence of Chinese *Cordyceps*. The increased microbial diversity might result in higher antibiosis and soil fungistasis, which might have negative effects on the development of *O. sinensis*, and ultimately suppress the occurrence of Chinese *Cordyceps*.

In terms of the composition of microbial taxa, at the bacterial phylum level, similar patterns were observed among all sampling sites (Figure 4), with *Proteobacteria*, *Acidobacteria*, and *Verrucomicrobia* being the most abundant bacterial phyla, which is in accordance with bacterial research on the habitat soil in Tibet [24]. For fungi, although *Ascomycota* and *Basidiomycota* were the most abundant fungal phyla among all sites, a perceived enrichment of other fungal phyla especially existed in site A. Coincidentally, fungal research on the soil adhering to the surface of the membrane covering Chinese *Cordyceps* also showed a predominance (approximately 90% in relative abundance) of other fungal phyla (unclassified fungi) [25]. Therefore, our study further indicates that some other unclassified fungi might be positively related to the occurrence of Chinese *Cordyceps*, and their role should be clarified in future research. At the OTU level, NMDS showed that both bacterial and fungal β-diversities were different for each sampling site, and the samples within each site were obviously grouped closely. At a genus level, the top 40 genera of soil bacterial and fungal communities (Figure 3) differed at different sites. Thus, the soil microbial taxonomic composition was different at both the OTU and genus level among the sites. Beside the top 40 genera above, 20 bacterial and 24 fungal OTUs were significantly enriched in the null Chinese *Cordyceps* group (site C) based on LDA (with LDA scores higher than 3.5). Among them, most of the bacteria may be responsible for the removal of anthropogenic compounds (*Rhodocyclales*) [41], autotrophic bacteria (*Chloroflexi*) [42], or anaerobic bacteria (*Methanobacteria*) [43]. The presence of those bacteria indicates that the soil habitat at site C might be not friendly to the heterotrophic and anaerobic Chinese *Cordyceps*. The fungi belonging to *Helotiales* can decrease during larval development and increase in the survival and fecundity rate of *Lepidoptera* [44]. Thus, the enriched *Helotiales* at site C might be a benefit for the *Thitarodes* insect and indirectly suppress the occurrence of Chinese *Cordyceps*. Conclusively, the analysis of microbial taxa indicates that the microbial composition in the habitat soil varied among different sampling sites and might be closely related to the occurrence of Chinese *Cordyceps*.

In the natural environment, *O. sinensis* first colonizes in alpine plant roots and is transferred to *Thitarodes* larvae through feeding behavior [45]. The occurrence of Chinese *Cordyceps* on *Thitarodes* larvae is dominated by *O. sinensis* and accompanied by the growth of various microorganisms. Therefore, some scholars have proposed that Chinese *Cordyceps* should be studied as a unified microbial ecosystem. Various microorganisms have been identified from natural Chinese *Cordyceps* [11,25], and even sparked controversy regarding the anamorph of Chinese *Cordyceps*. Zhu et al. found that *Paecilomyces hepiali* coexisted with *Hirsutella sinensis* [40] (the most approved anamorph of Chinese *Cordyceps* [1]) in natural Chinese *Cordyceps*, and even underwent dynamic changes with the maturation process of Chinese *Cordyceps*. They proved that a combined infection of *Paecilomyces hepiali* and *Hirsutella sinensis* can significantly improve the infection efficiency [46], suggesting that the synergistic infection effect of multiple microbiota may be an important link in the occurrence of Chinese *Cordyceps*. In this study, *Cordyceps*-related fungi (*Ophiocordycipitaceae*, *Clavicipitaceae*, and *Cordycipitaceae*) [35] were commonly detected in minor abundance among all detected sample sites (Figure 7). In the soil, most of the entomogenous fungi, including *Cordyceps*-related fungus and *O. sinensis*, mostly prefer to colonize in plant roots and derive nutrition from plant sources in the absence of insect hosts [45]. Thus, the detected relative abundance of these three families in this study was extremely low; however, despite that, *Ophiocordycipitaceae* and *Clavicipitaceae* showed a preference in sites A and B. These findings indicate that the soil conditions of sites A and B might be better for the survival of *Cordyceps*-related fungi, and several *Cordyceps*-related fungal genera, such as *Metarhizium*, *Pochonia*, *Cordyceps*, *Engyodontium*, *Chaunopycnis*, *Elaphocordyceps*, *Haptocillium*, *Polycephalomyces*, *Purpureocillium*, and *Tolypocladium*, might play a positive role in the occurrence of Chinese *Cordyceps*. Nevertheless, *O. sinensis* was absent among all of the soil samples in this study. In order to avoid disturbance of the physicochemical and microbial analysis of the soil, we had removed the roots before analysis, and the *O. sinensis* might have been removed along with the roots, which would have resulted in its absence. Thus, plants are the essential medium for the occurrence of Chinese *Cordyceps*, not only for providing food for host *Thitarodes* larvae, but also for providing the living microhabitat for *O. sinensis* before encountering the host *Thitarodes* larvae. These findings indicate that the factors of rhizosphere, such as plant roots and *Cordyceps*-related fungi, might play a role in the occurrence of Chinese *Cordyceps*, while their definite role should be proved through an infection trial in future study.

### 4.3. Co-Occurrence Interactions of Soil Microbial Community

Besides the above-mentioned microbial diversity and composition, for each microhabitat, the microbiota establishes a complicated community with interactions among microbial species, including a range of complex positive (commensalism, mutualism) and negative (amensalism, parasitism or predation, and competition) interactions occurring among different microbial species [47]. The network analysis of microbial co-occurrence patterns, especially showing positive or negative correlations, can provide a new perspective to investigate the structure of complex microbial communities, potential microbial interactions, and their ecological roles [48,49]. The occurrence of Chinese *Cordyceps* is actually the outcome of the infection, colonization, and growth of an entomogenous fungus (*O. sinensis*) on host insects living in the soil. Thus, the soil ecological status, including the correlations of microbiomes in the habitat [47], would influence the infection process of *O. sinensis*.

In order to investigate the intra-kingdom correlations among the predominant microbial genera, we carried out an intra-kingdom network analysis based on the positive and negative correlations among the 40 most abundant bacterial and fungal genera of the soil microbial community (Table 5). Co-occurrence patterns show that the network compositions substantially differed among different sites and each network had a distinct set of module hubs and connectors (Figure 8). For the bacterial community, there were more positive correlations and average degrees in the *Cordyceps* group (sites A and B), while this was reversed in the fungal community, with the most in the null *Cordyceps* group (site C). The findings indicate that a closer correlation of the bacterial community might help for the colonization of *O. sinensis* at sites A and B, while antagonism might exist between *O. sinensis* and the other fungal genera, and these fungal genera were positively correlated and might synergistically suppress the occurrence of Chinese *Cordyceps* at site C.

Previous studies revealed that dysbiosis in the bacterial kingdom and extensive synergistic networks in the fungal kingdom may enhance colonization by certain fungal families (such as pathogenic fungus) in gut microbiome [50,51]. Thus, the inter-kingdom correlations might also influence the occurrence of Chinese *Cordyceps.* Inter-kingdom network analysis was performed based on the positive and negative correlations among the different bacterial and fungal families in the soil microbial community. Among all the families, the co-occurrence analysis showed that the number of positive correlations decreased and negative correlations increased for sites A, B, and C, and accordingly, the ratios of positive and negative correlations decreased and were close to 1 at site C (site A: 2.71; site B: 1.54; site C: 1.16), i.e., for the null *Cordyceps* group, there was little difference between the number of positive and negative correlations. Thus, it can be speculated that the occurrence rate of Chinese *Cordyceps* might be negatively correlated with the stability of the correlation state (equilibrium between positive and negative correlations) of the soil habitat. Furthermore, the average degree of the *Cordyceps* group is higher than the null *Cordyceps* group, indicating that a higher level of inter-kingdom correlations might be helpful for the occurrence of Chinese *Cordyceps*. In addition, this study found that at site A, the positive correlations with the *Cordyceps*-related fungal family (*Cordycipitaceae*) were enriched. This finding indicates that some other microbes might be especially positive for the *Cordyceps*-related fungal family and might assist in the occurrence of Chinese *Cordyceps*. Further study is needed to seek out and verify the related microbial species in rhizospheres especially positive correlated with *O. sinensis*, to ultimately promote the occurrence of Chinese *Cordyceps.*

Thus, co-occurrence ecological relationships may be important for the occurrence of Chinese *Cordyceps*, and it is necessary to further prove the speculations proposed in this study and keep the right balance of biological interactions to increase the occurrence rate of Chinese *Cordyceps* in artificial cultivation.

## 5. Conclusions

This study is the first attempt to comprehensively analyze the physicochemical and microbial parameters in the habitats of Chinese *Cordyceps* with an emphasis on their influence on its occurrence. Soil physicochemical parameters, including EOC, SOC, HAC, HMC, and pH, might be negatively related to the occurrence of Chinese *Cordyceps*. Several soil physicochemical parameters (pH, NH_4_^+^-N/NO_3_^−^-N, SOC, HMC, HAC, APO, APH, and MBC) and microbial properties could interact and mix together, which might affect the occurrence of Chinese *Cordyceps*. Microbial community analysis revealed that a low level of bacterial and fungal diversity was suitable for the occurrence of Chinese *Cordyceps*, and soil microbial composition in the habitat soil varied among different sampling sites and might be closely related to the occurrence of Chinese *Cordyceps* (e.g., some unclassified fungi). Intra-kingdom network revealed that a closer correlation of the bacterial community might help in the occurrence of Chinese *Cordyceps*, while a closer correlation of the fungal community might suppress it. Inter-kingdom network revealed that the occurrence rate of Chinese *Cordyceps* might be negatively correlated with the stability of the correlation state (equilibrium between positive and negative correlations) of the soil habitat. Thus, our analysis shows that co-occurring ecological relationships may be important for the occurrence of Chinese *Cordyceps*, and it is necessary to keep the right balance of biological interactions to increase the occurrence rate in artificial cultivation.

Conclusively, both soil physicochemical and microbial parameters in the habitats could be related with the occurrence of Chinese *Cordyceps* and our study could help in gaining a better understanding of the occurrence of Chinese *Cordyceps* and provide useful information for conservation and artificial cultivation of this valuable fungus-larva symbiote. However, the structure of the microbial community and the correlations in soil are complicated and vary greatly among different regions and under different stresses [52], thus it is necessary to conduct an extensive and systematic investigation of the habitat microbiome in future studies, and the definite role of the microbial community and the correlations should be confirmed by further experiments. Furthermore, in artificial-cultivated implication, the manipulation of soil microbial communities is difficult to achieve, and the present study also provides a clue that the control of physicochemical factors might aid the realization.

## Figures and Tables

**Table 1:** Information of sampling in this study.

Parameters	Site A	Site B	Site C
Latitude	29°36′09.6″ N	29°35′49.6″ N	29°36′11.0″ N
Longitude	94°36′23.1″ E	94°36′12.0″ E	94°36′19.0″ E
Elevation	4166 m	4170 m	4173 m
Density of *Thitarodes* larvae	50 larva/m^2^	70 larva/m^2^	75 larva/m^2^
Density of Chinese *Cordyceps*	5 Chinese *Cordyceps*/m^2^	1 Chinese *Cordyceps*/m^2^	0
Occurrence rate	10.00%	1.40%	0
Sampling time	Mid-July 2016	Mid-July 2016	Mid-July 2016
Sampling depth of the soil	10–20 cm	10–20 cm	10–20 cm

**Table 2:** Physicochemical properties of soil samples at each site.

Physicochemical Property	Chinese *Cordyceps* Group		Null Chinese *Cordyceps* Group
	Site A	Site B	Site C
SWC (%)	41.0 ± 0.4	40.9 ± 0.9	38.3 ± 0.7 ^##,@@^
pH	5.37 ± 0.18	5.41 ± 0.12	7.37 ± 0.18 ^##,@@^
NH_4_^+^-N (mg/kg)	85.15 ± 2.79	106.14 ± 6.43 **	71.21 ± 5.53 ^#,@@^
NO_3_^−^-N (mg/kg)	6.06 ± 0.48	27.11 ± 1.94 **	4.41 ± 0.61 ^#,@@^
NH_4_^+^-N/NO_3_^−^-N	14.11 ± 0.88	3.93 ± 0.33 **	16.549 ± 3.73 ^@@^
APH (mg/kg)	20.96 ± 0.84	91.57 ± 2.43 **	61.66 ± 2.95 ^##,@@^
APO (mg/kg)	202.00 ± 6.16	298.00 ± 3.87 **	277.60 ± 5.18 ^##,@@^
EOC (mg/kg)	19.07 ± 0.93	24.17 ± 1.07 **	25.03 ± 1.55 ^##^
TN (%)	5.31 ± 0.39	5.64 ± 0.33	5.25 ± 0.22
MBC (mg/kg)	1029.14 ± 21.27	2352.61 ± 135.21 **	1352.47 ± 35.6 ^##,@@^
SOC (g/kg)	18.90 ± 0.99	23.48 ± 1.85 *	25.69 ± 1.58 ^##^
DOC (g/kg)	0.90 ± 0.10	1.58 ± 0.12 **	1.10 ± 0.11^#,@@^
HEC (g/kg)	23.14 ± 1.10	30.92 ± 1.00 **	27.24 ± 1.74 ^#,@^
FAC (g/kg)	13.12 ± 0.78	16.7 ± 1.69 *	13.71 ± 1.69 ^@^
HAC (g/kg)	10.38 ± 0.62	15.45 ± 1.00 **	15.30 ± 1.92 ^#^
HMC (g/kg)	86.43 ± 1.08	120.34 ± 4.13 **	120.36 ± 2.38 ^##^

SWC: Soil water content; NH_4_^+^-N/NO_3_^−^-N: the ratio of NH_4_^+^-N compare to NO_3_^−^-N; APH, available phosphorus; APO, available potassium; EOC, easily oxidizable organic carbon; TN, total nitrogen; MBC, microbial biomass carbon; SOC, soil organic carbon; DOC, dissolved organic carbon; HEC, extractable humus carbon; FAC, fulvic acid carbon; HAC, humic acid carbon; HMC, humin carbon; * *p* < 0.05 compared to site A; ** *p* < 0.001 compared to site A; ^#^ *p* < 0.05 compared to site A; ^##^ *p* < 0.001 compared to site A; ^@^ *p* < 0.05 compared to site B; ^@@^ *p* < 0.001 compared to site B.

**Table 3:** Diversity indices of soil microbial communities based on 16S rRNA and internal transcribed spacer (ITS) genes.

Classified	Sample Site	Number of Sequences	Number of OTUs	Shannon	Simpson	Chao1
Bacteria	Site A	42,129 ± 7501	3648 ± 334	9.38 ± 0.35	0.99 ± 0.00	4172 ± 380.85
	Site B	36,130 ± 9977	3437 ± 514	9.22 ± 0.19	0.99 ± 0.00	4221.6 ± 211.97
	Site C	42,276 ± 6043	4233 ± 377 *^,#^	9.62 ± 0.19^#^	1.00 ± 0.00^#^	4875.6 ± 279.04 *^,#^
Fungi	Site A	21,629.6 ± 9927	740 ± 140	4.9 ± 1.02	0.87 ± 0.06	769.4 ± 183.73
	Site B	150,145 ± 4308	711 ± 130	6.24 ± 0.44 *	0.95 ± 0.03 *	806 ± 90.02
	Site C	14,621 ± 6460	681 ± 84	6.16 ± 0.66 *	0.95 ± 0.02 *	780 ± 79.03

* *p* < 0.05 compared to site A; ^#^ *p* < 0.05 compared to site B. OTU, operational taxonomic unit.

**Table 4:** Dissimilarity comparison of soil microbial community structure among all sampling sites. ANOSIM: analysis of similarities, MRPP: multi-response permutation procedure.

Classified	ANOSIM		Adonis			MRPP		
	*R*	*P*	*F*	*R* ^2^	*P*	Delta (δ)	Effect Size (A)	*P*
Bacteria	0.564	0.001	3.910	0.395	0.001	0.090	0.131	0.004
Fungi	0.545	0.001	2.697	0.310	0.001	0.318	0.106	0.001

**Table 5:** Intra-kingdom and inter-kingdom analysis of soil microbial communities.

	Classified	Site	Average Degree	Positive Correlations	Negative Correlations	Positive/Negative	Nodes
Intra-kingdom analysis based on top 40 genera	Bacteria	A	1.143	16	4	/	35
		B	1.256	13	6	/	31
		C	0.571	4	4	/	28
	Fungi	A	0.414	6	0	/	29
		B	0.667	7	4	/	33
		C	0.733	11	0	/	30
Inter-kingdom analysis of all family levels	Bacteria and Fungi	A	4.241	257	95	2.71	166
		B	5.383	243	158	1.54	149
		C	3.831	182	157	1.16	177

## Supplementary Materials

Supplementary materials are available online at https://www.mdpi.com/2076-2607/7/9/284/s1. Table S1: OTU for the analysis of fungi and bacteria. Table S2: Result of Mantel test for the test of influence of soil physicochemical properties on soil microbial community. Table S3 and Table S4: Relative compositions of soil bacterial and fungal communities at the phylum, class, order, family, and genus level.
