# Supplementary material for: Diversity and Co-Occurrence Patterns of Soil Bacterial and Fungal Communities of Chinese Cordyceps Habitats at Shergyla Mountain, Tibet: Implications for the Occurrence
Source: Microorganisms. 2019 Aug 22;7(9):284. doi: 10.3390/microorganisms7090284 (PMC6780579; doi:10.3390/microorganisms7090284)
Supplement: Supplementary file 1 [file microorganisms-07-00284-s001.zip › Table S2-Result of Mantel test for the test of influence of soil physicochemical properties on soil microbial community.doc]

**Table S2 Result of Mantel test for the test of influence of soil physicochemical properties on soil microbial community**

| **Physicochemical properties** | **Bacterial community** | |  | **Fungi community** | |
| --- | --- | --- | --- | --- | --- |
|  | **Mantel statistic r** | **Significance (*P*)** |  | **Mantel statistic r** | **Significance (*P*)** |
| PH | 0.4117 | 0.002 |  | 0.3908 | 0.003 |
| SWC | 0.2273 | 0.068 |  | 0.2601 | 0.011 |
| NH_4_^+^-N | 0.1956 | 0.071 |  | 0.3076 | 0.005 |
| NO_3_^-^-N | 0.1608 | 0.101 |  | 0.3262 | 0.005 |
| NH_4_^+^-N/NO_3_^-^-N | 0.3552 | 0.009 |  | 0.3982 | 0.003 |
| APH | 0.2861 | 0.009 |  | 0.6504 | 0.001 |
| APO | 0.2662 | 0.016 |  | 0.6264 | 0.001 |
| EOC | 0.2105 | 0.06 |  | 0.5624 | 0.001 |
| MBC | 0.2292 | 0.034 |  | 0.4386 | 0.001 |
| SOC | 0.3263 | 0.006 |  | 0.5652 | 0.001 |
| DOC | 0.1189 | 0.152 |  | 0.4345 | 0.002 |
| HEC | 0.1733 | 0.071 |  | 0.5527 | 0.001 |
| HAC | 0.2342 | 0.015 |  | 0.5389 | 0.001 |
| HMC | 0.246 | 0.019 |  | 0.6221 | 0.001 |
| FAC | 0.1354 | 0.17 |  | 0.1376 | 0.105 |
| TN | 0.0002535 | 0.45 |  | -0.1933 | 0.976 |
